# Characterizing topological properties in 2D textures with magnetic structure tensors

**DOI:** 10.1016/j.isci.2025.112592

**Published:** 2025-05-05

**Authors:** Seong Min Park, Tae Jung Moon, Gyunghun Yu, Han Gyu Yoon, Jun Woo Choi, Hee Young Kwon, Changyeon Won

**Affiliations:** 1 Department of Physics, Kyung Hee University, Seoul 02447, South Korea; 2 Center for Spintronics, Korea Institute of Science and Technology, Seoul 02792, South Korea

**Keywords:** Engineering, Materials science, Physics

## Abstract

The spin structures, including magnetic skyrmions, have attracted great attention due to their interesting properties caused by their topology. We introduce a magnetic structure tensor as a powerful tool for characterizing topological properties in two-dimensional spin textures. We derived an order parameter from the two orthogonal components of the magnetic structure tensor. The order parameter effectively analyzes the structures of the spin patterns and identifies topological defects such as the end or branching points of linear chiral structures and magnetic skyrmions, as a pole or multipoles composed of topological quantized charges. We present several spin configurations interpreted as topological monopoles, dipoles, and quadrupoles formed by the combination of these topological poles. This approach provides valuable insights into defect distribution and interactions, validated through both simulations and experimental data. Moreover, the approach can be generally applied for effective analysis of self-organized patterns such as fingerprints.

## Highlights

• The magnetic structure tensor is introduced to characterize 2D chiral magnetic textures
• An order parameter is defined to identify the point defects in the magnetic textures
• The approach is validated through simulations, experiments, and generalized patterns

## Graphical abstract

**Figure:**
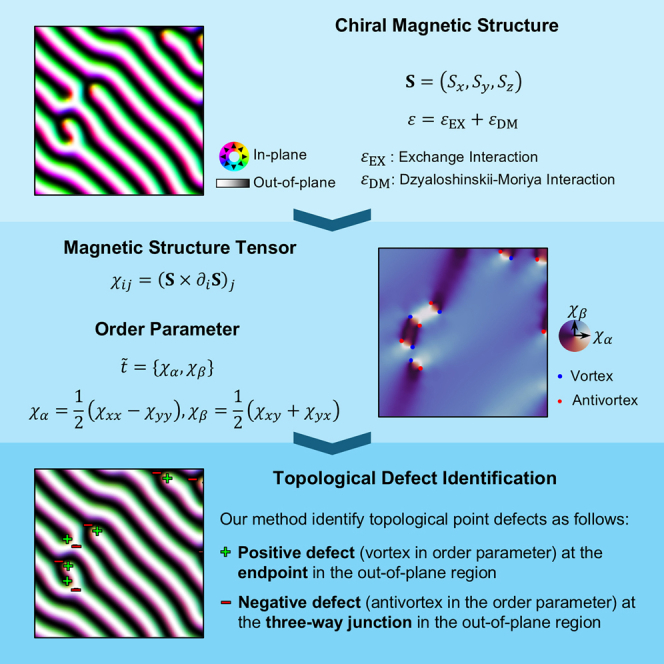

## Introduction

In condensed matter physics, topology has become a powerful framework for understanding and classifying various phenomena, such as dislocations in crystal lattices and phases of matter.^1^^,^^2^ Topology focuses on properties of space that remain unchanged under continuous deformations, providing a robust framework for classifying and understanding the behavior of complex systems. In magnetism, topological concepts are essential for studying the stability and dynamics of magnetic textures like domain walls, vortices, and skyrmions.^3^^,^^4^^,^^5^^,^^6^ Magnetic skyrmions have gained significant attention recently due to their topological properties and potential applications in spintronics.^7^^,^^8^^,^^9^^,^^10^^,^^11^^,^^12^^,^^13^^,^^14^ These structures are characterized by topological invariants, such as the winding number and the skyrmion number.^15^^,^^16^ These invariants are conserved quantities that confer remarkable stability against perturbations.^17^

A promising application of topology is in describing defects in underlying orders. These defects are determined by identifying the homotopy class of a closed contour around the defects.^1^^,^^18^^,^^19^ To analyze defects using homotopy, the spin configuration around a defect is mapped onto a parameter space V and this mapping is examined over a closed contour, $S^{r}$, where r represents the dimension of the contour. The homotopy class is then characterized by the homotopy group, $\pi_{r}\left(V\right)$, which describes the topological defects. For example, the vortices and antivortices in the two-dimensional XY spin model ($V=S^{1}$) are classified by identifying the homotopy class of one-dimensional loop ($\pi_{1}\left(S^{1}\right)$).^19^ In the three-dimensional Heisenberg spin model ($V=S^{2}$), the Bloch point is defined by the homotopy class of the surface surrounding the Bloch point ($\pi_{2}\left(S^{2}\right)$).^5^ These definitions are valid only when the homotopy groups are non-trivial, providing a rigorous mathematical framework to classify and understand defects in various magnetic textures.

Building on these concepts, we investigate two-dimensional noncollinear magnetic textures using a Hamiltonian model that includes exchange and the Dzyaloshinskii-Moriya (DM) interactions.^20^^,^^21^ The competition between these interactions gives rise to a variety of topological structures, including helical spin textures and skyrmions.^22^^,^^23^^,^^24^ These structures are topologically protected, meaning that transitioning from one structure to another requires discontinuous deformation. For instance, a well-aligned stripe pattern—a typical ground state structure, is topologically protected, so any dislocations or defects render it distinct from its original configuration. However, directly identifying the homotopy class to determine these defects is challenging due to the trivial nature of the homotopy group ($\pi_{1}\left(S^{2}\right)=0$).^18^

To address this challenge, we present a novel approach for defining topological defects in two-dimensional magnetic textures by introducing a magnetic structure tensor. The magnetic structure tensor is defined by the cross product of spins and their spatial derivatives, effectively capturing the intricate nature of these textures. Based on the magnetic structure tensor, we define an order parameter that represents the underlying order of the magnetic textures. Topological defects are identified by classifying the homotopy classes of the order parameters, analogous to how vortices and antivortices are identified in the two-dimensional XY model. We provide examples of spin configurations and corresponding topological defects, carefully selected to illustrate various cases, such as topological dipoles and quadrupoles. These examples demonstrate the robustness and versatility of our approach. Furthermore, we expand our methodology for general pattern analysis, such as fingerprint analysis, since our method effectively characterizes the orientation of patterns and identifies the topological defects in the patterns.

## Results and discussion

### Theoretical framework

**Figure 1:**
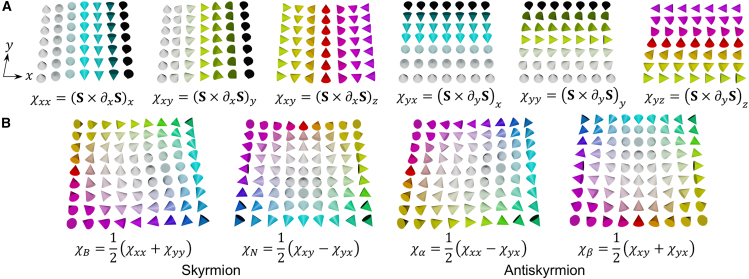
Magnetic textures corresponding to each component of the magnetic structure tensor defined in our study (A) Magnetic textures corresponding to each $\chi_{ij}$. The cones refer to the spin direction. The color of the cones indicates the in-plane spin directions, while the black-white contrast indicates the out-of-plane component of the spins. (B) The magnetic textures corresponding to the Bloch-type ($\chi_{B}$), Néel-type ($\chi_{N}$) chiral magnetic structures, and the antiskyrmion-like structures ($\chi_{\alpha }$ and $\chi_{\beta }$).

Our interest lies in the two-dimensional Heisenberg spin model, which represents a three-dimensional normalized vector field on a two-dimensional space. Due to the normalization condition, the variation of the spin over the space is restricted to rotations of the spins without changing their magnitude. To capture this behavior, we define the magnetic structure tensor, $\chi_{ij}$, as the rotation of the spin vector S along the spatial directions. The magnetic structure tensor can be expressed as the cross product of the spin vector and its spatial derivative:

(Equation 1) $$\chi_{ij}={\left(S\times \left(\partial_{i}S\right)\right)}_{j}$$

where i∈{x,y} represents the spatial directions, and j∈{x,y,z} represents the Cartesian coordinate components of spin rotation. Figure 1A illustrates the magnetic textures corresponding to each component of $\chi_{ij}$. As spatial derivatives are used in the magnetic structure tensor formula, the tensor characterizes how the spin vector S changes spatially at a given location. In discrete lattice models, the tensor corresponds to the expression of form $S\times S_{\text{neighbor}}$. In this discrete formulation, the index i in Equation 1 denotes the direction from the reference spin S to its nearest neighbor $S_{\text{neighbor}}$. The continuum form of the chiral tensor, involving spatial derivatives, is valid when $\left\|S_{\text{neighbor}}-S\right\|\ll \left\|S\right\|$, i.e., when the spin field varies smoothly and the continuum approximation holds. Under this condition, the spin texture can be treated as effectively continuous, and the influence of discrete lattice features such as lattice symmetry becomes negligible.

Since the magnetic structure tensor is intrinsically linked to the derivatives of the spin field, it is suitable for analyzing the exchange interaction and DM interaction. In real magnetic systems, other contributions—such as external magnetic fields or magnetocrystalline anisotropy—may also influence spin configurations. Note that our energetic analysis, based on the magnetic structure tensor, is inherently limited to systems dominated by exchange and DM interactions. Nevertheless, the defect classification method based on the magnetic structure tensor (described in the next section) remains valid even in more complex systems. This robustness stems from the fact that the topological classification of defects is insensitive to the detailed local profiles. Therefore, as long as the system is dominated by exchange and DM interactions, the overall defect classification remains unaffected by additional energy terms that modify the spin profiles.

Using the magnetic structure tensor, we investigate a system modeled by a Hamiltonian that includes both exchange and DM interactions. The magnetic structure tensor effectively captures these interactions. The general expression for the exchange interaction energy density is given by:

(Equation 2) $$\varepsilon_{\text{EX}}=\sum_{ij}J_{ij}\partial_{i}S\cdot \partial_{j}S=\sum_{ijk}J_{ij}\chi_{ik}\chi_{jk}=\text{Tr}\left(\chi^{T}J\chi \right)$$

where the $J_{ij}$ is the exchange interaction parameter. The DM interaction energy density can also be expressed in terms of the magnetic structure tensor:

(Equation 3) $$\varepsilon_{\text{DM}}=-\sum_{i}\;{\vec{D}}_{i}\cdot \left(S\times \partial_{i}S\right)=-\sum_{ij}\;D_{ij}\chi_{ij}=-\text{Tr}\left(\chi^{T}D\right)$$

where the ${\vec{D}}_{i}$ is the DM vector along the i-axis.

Specifically, the components $\chi_{xx}$, $\chi_{xy}$, $\chi_{yx}$, and $\chi_{yy}$ represent chiral magnetic structures. To analyze the magnetic structure tensor $\chi_{ij}$ according to its rotational symmetry, we decompose it into a set of matrices {$m_{q}$}, consisting of the identity matrix $I=\left(\begin{array}{cc} 1 & 0\\ 0 & 1 \end{array}\right)$, the Levi-Civita matrix $\epsilon =\left(\begin{array}{cc} 0 & 1\\ -1 & 0 \end{array}\right)$, and the Pauli matrices $\sigma_{z}=\left(\begin{array}{cc} 1 & 0\\ 0 & -1 \end{array}\right)$ and $\sigma_{x}=\left(\begin{array}{cc} 0 & 1\\ 1 & 0 \end{array}\right)$:

(Equation 4) $$\left(\begin{array}{cc} \chi_{xx} & \chi_{xy}\\ \chi_{yx} & \chi_{yy} \end{array}\right)=\chi_{B}I+\chi_{N}\epsilon +\chi_{\alpha }\sigma_{z}+\chi_{\beta }\sigma_{x}$$

The decomposed components are found by $\frac{1}{2}\text{Tr}\left(m_{q}\left(\begin{array}{cc} \chi_{xx} & \chi_{xy}\\ \chi_{yx} & \chi_{yy} \end{array}\right)\right)$:

(Equation 5) $$\chi_{B}=\frac{1}{2}\left(\chi_{xx}+\chi_{yy}\right),\chi_{N}=\frac{1}{2}\left(\chi_{xy}-\chi_{yx}\right),\chi_{\alpha }=\frac{1}{2}\left(\chi_{xx}-\chi_{yy}\right),\chi_{\beta }=\frac{1}{2}\left(\chi_{xy}+\chi_{yx}\right)$$

Here, $\chi_{B}$ and $\chi_{N}$ represent the Bloch-type and Néel-type chiral magnetic structures, respectively, while $\chi_{\alpha }$ and $\chi_{\beta }$ correspond to antiskyrmion structures.^12^^,^^25^ Figure 1B illustrates the magnetic textures corresponding to these components.

It should be noted that the skyrmion-type components, $\chi_{B}$ and $\chi_{N}$, are invariant under rotations, while the antiskyrmion-type components, $\chi_{\alpha }$, and $\chi_{\beta }$, rotate by twice the rotation angle defined by $R\left(\theta \right)=\left(\begin{array}{cc} \cos\;\theta & -\sin\;\theta \\ \sin\;\theta & \cos\;\theta \end{array}\right)$. In other words, when the system rotates by an angle θ, we have:

(Equation 6) $$\begin{array}{ll} \chi_{B}^{\prime } & =\chi_{B}\\ \chi_{N}^{\prime } & =\chi_{N}\\ \chi_{\alpha }^{\prime } & =\chi_{\alpha }\;\cos\;2\theta -\chi_{\beta }\;\sin\;2\theta \\ \chi_{\beta }^{\prime } & =\chi_{\alpha }\;\sin\;2\theta +\chi_{\beta }\;\cos\;2\theta \end{array}$$

This follows from the fact that the magnetic structure tensor transforms under rotation as $\left(\begin{array}{cc} \chi_{xx}^{\prime } & \chi_{xy}^{\prime }\\ \chi_{yx}^{\prime } & \chi_{yy}^{\prime } \end{array}\right)=R\left(\theta \right)\left(\begin{array}{cc} \chi_{xx} & \chi_{xy}\\ \chi_{yx} & \chi_{yy} \end{array}\right)R^{T}\left(\theta \right)$. From the Equation 4, we write

(Equation 7) $$\chi_{B}^{\prime }I+\chi_{N}^{\prime }\epsilon +\chi_{\alpha }^{\prime }\sigma_{z}+\chi_{\beta }^{\prime }\sigma_{x}=R\left(\theta \right)\left[\chi_{B}I+\chi_{N}\epsilon +\chi_{\alpha }\sigma_{z}+\chi_{\beta }\sigma_{x}\right]R^{T}\left(\theta \right)$$

and using relations

(Equation 8) $$\begin{array}{ll} R\left(\theta \right)IR^{T}\left(\theta \right) & =I\\ R\left(\theta \right)\epsilon R^{T}\left(\theta \right) & =\epsilon \\ R\left(\theta \right)\sigma_{z}R^{T}\left(\theta \right) & =\cos\;2\theta \sigma_{z}+\sin\;2\theta \sigma_{x}\\ R\left(\theta \right)\sigma_{x}R^{T}\left(\theta \right) & =-\sin\;2\theta \sigma_{z}+\cos\;2\theta \sigma_{x} \end{array}$$

we recover the stated transformation for the skyrmion-type components and the antiskyrmion-type components.

Utilizing the magnetic structure tensor, we demonstrate topological defect identification for a typical system with isotropic exchange interaction and Bloch-type DM interaction. The Hamiltonian parameters are given by $J_{xx}=J_{yy}=J,J_{xy}=J_{yx}=0,{\vec{D}}_{x}=D\hat{y},{\vec{D}}_{y}=-D\hat{x}$, where J and D refer to the strength of exchange interaction and DM interaction, respectively. In this system, the energy density is expressed as:

(Equation 9) $$\varepsilon =J\left(\chi_{xx}^{2}+\chi_{xy}^{2}+\chi_{xz}^{2}+\chi_{yx}^{2}+\chi_{yy}^{2}+\chi_{yz}^{2}\right)-D\left(\chi_{xx}+\chi_{yy}\right)$$

This can be rewritten in terms of the reorganized components as:

(Equation 10) $$\varepsilon =J\left(2\chi_{B}^{2}+2\chi_{N}^{2}+2\chi_{\alpha }^{2}+2\chi_{\beta }^{2}+\chi_{xz}^{2}+\chi_{yz}^{2}\right)-2D\chi_{B}$$

Indeed, when energy is expressed as density, a straightforward local minimization typically leads to a trivial solution in which most terms vanish, leaving only the terms of $\varepsilon =2J\chi_{B}^{2}-2\chi_{B}$. This implies that the spins vary in every direction within the two-dimensional plane, analogous to the skyrmion core. However, this local minimization condition is insufficient to describe the entire two-dimensional spin configuration. In practice, the ground states of the system are well-aligned helical textures: the spins vary helically along one direction while remaining uniform along the perpendicular direction.^26^ Due to rotational symmetry, the orientation of the ground state alignment can be arbitrary. The energy minimization conditions of the ground states are given by: $\chi_{N}=\chi_{xz}=\chi_{yz}=0$ and $\chi_{\alpha }^{2}+\chi_{\beta }^{2}=\chi_{B}^{2}$. Thus, the energy is given by $\varepsilon =4J\chi_{B}^{2}-2D\chi_{B}$, and the energy density and chiral strength at the ground state are $\varepsilon_{0}=-\frac{D^{2}}{4J}$ and $\chi_{0}=\frac{D}{4J}$, respectively. The corresponding periodicity from the chiral strength is $\lambda_{0}=\frac{\pi }{\chi_{0}}$.

The magnetic structure tensor of the ground state satisfies the relation:

(Equation 11) $$\chi_{\alpha }^{2}+\chi_{\beta }^{2}=\chi_{0}^{2}$$

which implies that the $\chi_{\alpha }$ and $\chi_{\beta }$ lie on a circle of fixed radius $\chi_{0}$ in the $\left(\chi_{\alpha },\chi_{\beta }\right)$ plane. To capture the orientation of the magnetic texture, we define an order parameter:

(Equation 12) $$\tilde{t}=\left\{\chi_{\alpha },\chi_{\beta }\right\}$$

We use the tilde and curly brackets to emphasize that although the $\tilde{t}$ has two components, it is not a conventional vector because does not transform as ${\tilde{t}}^{\prime }=R\left(\theta \right)\tilde{t}$ under rotations. Indeed, as discussed earlier, the $\chi_{\alpha }$ and $\chi_{\beta }$ rotate with a double-angle dependence. Consequently, the order parameter $\tilde{t}$ reflects the orientation of the ground state magnetic texture, which exhibits a well-aligned helical pattern that recovers its structure after a rotation of π (rather than 2π). Finally, the topological point defects are classified by the homotopy group $\pi_{1}\left(S^{1}\right)$: the winding number of $\tilde{t}$ along a closed contour surrounding the point determines whether that point constitutes a topological defect, analogous to the identification of vortices and antivortices in the two-dimensional XY model.

Although our previous discussion focused on a system with Bloch-type DM interaction, our order parameter approach—including defect identification—remains valid for systems with Néel-type DM interaction or a combination of Néel-type and Bloch-type DM interactions. In fact, these DM interactions can be interpreted as a spin rotation about z axis relative to the Bloch-type DM interaction. In other words, the spin configurations under general DM interaction can be obtained by transforming the spins as:

(Equation 13) $$S^{*}=R\left(\theta \right)S,R\left(\theta \right)=\left(\begin{array}{ccc} \cos\;\theta & -\sin\;\theta & 0\\ \sin\;\theta & \cos\;\theta & 0\\ 0 & 0 & 1 \end{array}\right)$$

Here, we use the R(θ) notation (a 3×3 rotation matrix) to distinguish it from the 2×2 rotation matrix R(θ). For example, by rotating the spins by $\theta =\frac{\pi }{2}$, a Bloch-type spin configuration can be transformed into a Néel-type one. It is important to note that the spin rotation we discuss is distinct from the rotation of the entire system. A system (or coordinate) rotation is applied to the basis, affecting both the spin vectors and the spatial derivatives (e.g., $\partial_{i}$ transforms as well).

As the spins are rotated, the magnetic structure tensor transforms as $\chi_{ij}^{*}={\left(R\left(\theta \right)S\times \partial_{i}\left(R\left(\theta \right)S\right)\right)}_{j}$. Since the cross product transforms covariantly under rotations, we have $\chi_{ij}^{*}=R\left(\theta \right)\chi_{ij}$. From the Equation 4, this transformation is written as:

(Equation 14) $$\chi_{B}^{*}I+\chi_{N}^{*}\epsilon +\chi_{\alpha }^{*}\sigma_{z}+\chi_{\beta }^{*}\sigma_{x}=R\left(\theta \right)\left[\chi_{B}I+\chi_{N}\epsilon +\chi_{\alpha }\sigma_{z}+\chi_{\beta }\sigma_{x}\right]$$

Note that there are no z axis components in the decomposed expression, which is why we use the R(θ) notation. The decomposed components $\left(\chi_{B},\chi_{N}\right)$ and $\left(\chi_{\alpha },\chi_{\beta }\right)$ transforms with the same angle as the spin rotation. In conclusion, under a general DM interaction, the order parameter $\tilde{t}=\left\{\chi_{\alpha },\chi_{\beta }\right\}$ acquires only a phase shift of θ during the transformation:

(Equation 15) $$\begin{array}{cc} \chi_{\alpha }^{*} & =\cos\;\theta \chi_{\alpha }-\sin\;\theta \chi_{\beta }\\ \chi_{\beta }^{*} & =\sin\;\theta \chi_{\beta }+\cos\;\theta \chi_{\alpha } \end{array}$$

Thus, the defect identification—which classifies topological point defects by the winding of the order parameter $\tilde{t}=\left\{\chi_{\alpha },\chi_{\beta }\right\}$—remains unaffected by this transformation. This robustness confirms that our defect identification method remains valid even when the underlying DM interaction deviates from Bloch-type.

Additionally, another application of $\tilde{t}$ is to obtain an orientation field^27^^,^^28^^,^^29^ for the spin configurations. The orientation can be computed directly from $\tilde{t}$ using the relation $\theta_{O}=\frac{1}{2}\theta_{\tilde{t}}+\frac{\pi }{2}$, where $\theta_{O}$ and $\theta_{\tilde{t}}$ denote the angles of O and $\tilde{t}$, respectively. This orientation represents the ordering direction of the chiral spin structure.

### Topological point defects identification

**Figure 2:**
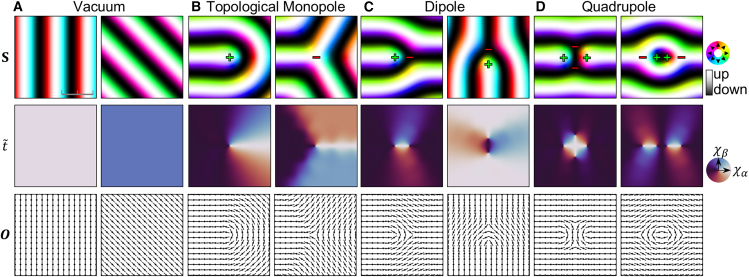
Topological point defects identification for magnetic structures (A–D) Visualizations of spin configurations S along with the corresponding order parameter field $\tilde{t}$ and the orientation field O for various topological defect configurations: (A) absence of topological defects (vacuum), (B) topological monopoles, (C) dipoles, and (D) quadrupoles. The colors in the spin configurations represent the in-plane spin components, while the black-white contrasts represent the out-of-plane spin component. The color in the order parameter visualizations represents the direction of the order parameter $\tilde{t}$. The ticks of the scale bar represent intervals of length πJ/D, which is the characteristic length scale of the system.

In this section, we demonstrate our topological point defect identification method by applying it to simulation results implemented on a square lattice under the Bloch-type Hamiltonian model with J=1 and D=0.1. Figure 2A illustrates the ground state spin configurations along with the corresponding order parameter $\tilde{t}$ and the orientation field O. The order parameter $\tilde{t}$ characterizes the magnitude and phase of the magnetic texture, whereas the orientation field O indicates the direction of alignment of the spin configurations. Due to the competition between exchange and DM interactions, the ground state spin structures form a stripe pattern.^24^ These helically varying structures yield uniform order parameter $\tilde{t}=\left\{\chi_{\alpha },\chi_{\beta }\right\}$ (Equation 12) with the magnitude of $\left|{\tilde{t}}_{0}\right|=\frac{D}{4J}$, which defines a characteristic length scale of the system. Consequently, the ground state spin configuration does not produce any topological defects in the $\tilde{t}$ field. Notably, the angles of $\tilde{t}$ for the first and second examples differ by 90°, even though the spin configurations differ by only 45°. This observation is consistent with the double-angle rotational behavior of the antiskyrmion-type components discussed earlier (see Equation 6). The orientation field O effectively captures the overall directionality of the magnetic texture, representing the alignment of the spin configuration.

Figure 2B presents typical examples of isolated topological point defects with opposite charges. In our work, we refer to these defects as “topological monopoles”. It is important to note that this nomenclature is not intended to imply the conventional concept of a magnetic monopole, which is defined via a net flux through a closed surface (as in Gauss’s law). Our topological monopoles are isolated topological point defects classified by the homotopy group the homotopy group $\pi_{1}\left(S^{1}\right)$. In other words, our defect classification focuses on the winding of the order parameter $\tilde{t}$ along a closed contour around the defect. In this classification, an endpoint in the out-of-plane spin region is identified as a topological monopole because $\tilde{t}$ winds once around the defect, yielding a topological charge of +1 in $\pi_{1}\left(S^{1}\right)$. Notably, the endpoint is commonly referred to as a half-skyrmion, carrying a charge of $+\frac{1}{2}$ in terms of the skyrmion number. For the three-way junction in the out-of-plane region, $\tilde{t}$ undergoes a single winding in the opposite direction (i.e., counterclockwise for a clockwise contour), corresponding to a topological charge of −1 in $\pi_{1}\left(S^{1}\right)$. Therefore, the endpoint and three-way junction in the out-of-plane spin region represent topological monopoles with positive and negative charges, respectively.

Based on these results, we examine further examples in which defects form dipoles or quadrupoles. Spin structures comprising a pair consisting of an endpoint and a three-way junction result in dipoles of point defects, as shown in Figure 2C. Notably, the order parameter $\tilde{t}$ for these structures can be enclosed within a uniform boundary. Analogous to the XY model, the order parameter defined within a uniform or periodic boundary contains either no topological defects or defects in pairs.^19^ Figure 2D illustrates a quadrupole configuration of topological defects, which can be generated by introducing a structural defect—such as a disconnection or an additional skyrmion—into the ground state spin configuration.

### Energetic analysis for topological defects

**Figure 3:**
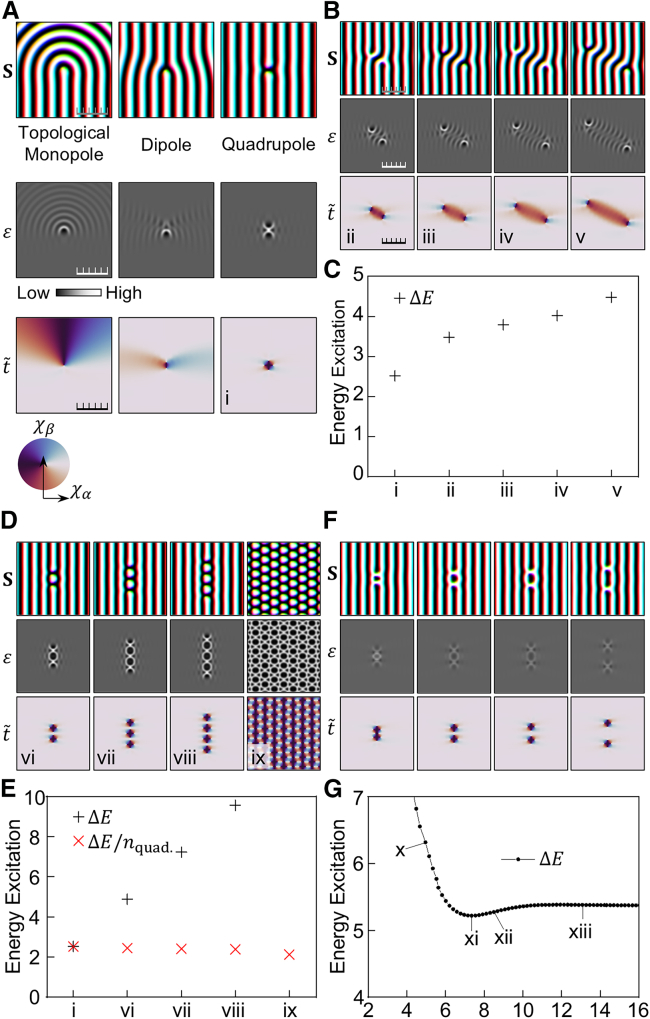
Energetic analysis for magnetic structures including topological point defects (A) The spin configuration S, local energy density ε, and the order parameter $\tilde{t}$ of topological monopole, dipole, and quadrupole. (B) S, ε, and $\tilde{t}$ of various magnetic structures whose topological defects form different quadrupoles. (C) The energy excitation ($\Delta E=\int \left(\varepsilon -\varepsilon_{0}\right)$), proportional to the exchange interaction constant, J. The indices ii–v refer to the spin configurations in (B), while the index i refers to the quadrupole spin configuration in (A). (D) S, ε, and $\tilde{t}$ of magnetic structures containing different numbers of quadrupoles: vi represents two quadrupoles, vii represents three, viii represents four, and ix represents a dense arrangement of quadrupoles. (E) The total energy excitation (ΔE) of the spin configurations along with the energy excitation per topological quadrupoles ($\Delta E/n_{\text{quad}.}$). (F) S, ε, and $\tilde{t}$ of magnetic structures containing compressed (x), released (xi), and elongated skyrmions (xii and xi). (G) The relation between energy excitation and the elongation (compression) of magnetic skyrmion. The *x* axis represents the vertical distance between the negative topological defects (three-way points), scaled by the characteristic length πJ/D. The ticks of the scale bars represent intervals of length πJ/D.

To further understand the behavior of topological defects, we perform an energetic analysis. Figure 3A displays the spin configurations for topological monopoles, dipoles, and quadrupoles, along with their respective local energy densities, ε, and the order parameter, $\tilde{t}$. As previously discussed, the endpoint spin structure exhibits a topological monopole. In this configuration, the curved structure extends so that energy excitations are distributed over a wide area rather than being localized at the point defect. The energy excitation is calculated as $\int_{0}^{R}\left(\varepsilon_{\text{monopole}}-\varepsilon_{0}\right)2\pi rdr\propto J\;\ln\left(\frac{D}{J}R\right)$, assuming a simple radially structured spin model given by $S_{x}=\sin\left(kr\right)\cos\left(\theta \right)$, $S_{y}=\sin\left(kr\right)\sin\left(\theta \right)$, and $S_{z}=\cos\left(kr\right)$, where $r=\sqrt{x^{2}+y^{2}}$ and θ=arctan(y/x). This logarithmic dependence implies that the energy excitation is not confined locally, emphasizing the delocalized nature of the energy in the topological monopole configurations. Similarly, the spin configuration with a topological dipole exhibits an extended energy distribution, indicating that the topological dipole is also delocalized. In contrast, the energy of the topological quadrupole is more localized and can be effectively confined within the ground state spin configuration. The local energy of the quadrupole spin configuration rapidly approaches the ground state value as the distance from the quadrupole increases, highlighting the confined nature of its energy distribution.

The energetic confinement of the quadrupole spin configuration enables a quantitative comparison of its energy with that of the ground state. Figure 3B displays spin configurations S along with the local energy density ε and order parameter $\tilde{t}$, where topological defects form different quadrupoles. The spin configurations cannot transition from one to another without disrupting the stripe pattern, indicating that they are topologically distinct. From configuration (ii) to (v), the topological defects occupy increasingly larger areas, leading to energy excitation in those regions, as reflected in ε. Consequently, a larger area of topological defects results in greater total energy excitation, as shown in Figure 3C. We confirm that the smallest quadrupole in Figure 3A is the most energetically stable among the quadrupoles. Figure 3D displays additional examples featuring multiple topological quadrupoles. As each quadrupole is introduced, a magnetic skyrmion appears in the spin configuration. A dense arrangement of topological quadrupoles corresponds to a dense lattice of magnetic skyrmions. As shown in Figure 3E, the total energy excitation shows a linear relationship with the number of introduced quadrupoles.

Furthermore, Figures 3F and 3G illustrate how the energy changes with the elongation (or compression) of the magnetic skyrmion^30^^,^^31^ in our system. The spin configurations labeled (x) to (xiii) in Figure 3F display skyrmions of different lengths. When the pinned spins are released, the skyrmion relaxes to its equilibrium length, making it impossible to systematically study the relationship between skyrmion length and energy. From a topological defect perspective, this process adjusts the distance between two quadrupoles. The compressed skyrmion (x) exhibits higher energy than the relaxed one (xi). Figure 3G shows the relationship between skyrmion elongation (or compression) and energy excitation, with the *x* axis representing the vertical distance between the negative defects, which serves as a measure of skyrmion length. Our results indicate that the spin configurations exhibit minimal energy excitation at the specific skyrmion length. In other words, there is an optimal separation between the two defect quadrupoles that minimizes energy. Once this distance becomes sufficiently large, the energy excitation no longer changes, indicating that the quadrupoles no longer interact significantly with each other.

### Distance analysis for the topological defects

**Figure 4:**
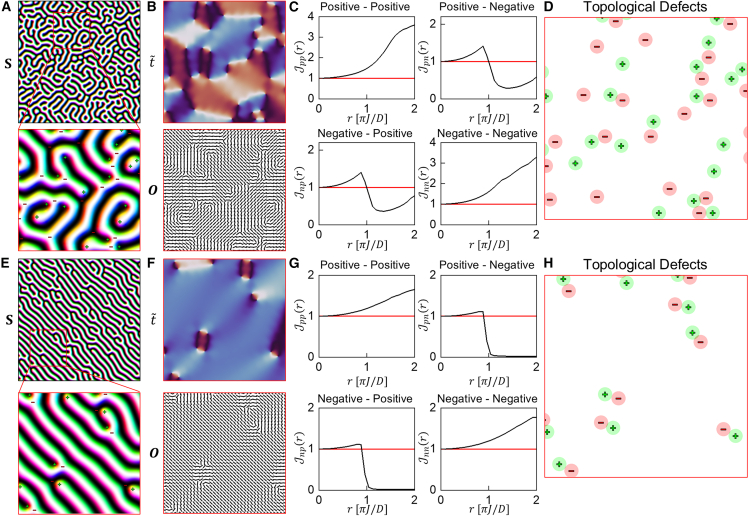
Pairwise distance analysis for randomly distributed topological point defects (A) The spin configuration containing numerous randomly generated topological defects. (B) The corresponding order parameter $\tilde{t}$ and orientation field O for the zoomed-in area. (C) The J-functions computed from the topological defects in (A). (D) The topological defects in the zoomed-in area. The circles indicate the effective area of each defect, with a radius of the characteristic length πJ/D. (E–H) The same analysis for a different spin configuration which contains fewer topological defects compared to the spin configuration in (A). The red edges of the images indicate that the images are focusing on the zoomed-in area.

Now, we perform a pairwise distance analysis for the topological defects, which are generated by a specific random process. Figure 4A shows a spin configuration obtained through a Monte Carlo simulated annealing process.^23^^,^^24^^,^^32^^,^^33^ During annealing, spontaneous symmetry breaking occurs, leading to the formation of a complex spin structure.^34^ In the final spin configuration, numerous topological defects are randomly distributed. As shown in Figure 4B, the order parameter $\tilde{t}$ and the orientation field O effectively highlight the alignment direction of the spin configuration while omitting the details of the helical structure.

To investigate the effective interaction between the topological defects, we employ the J-function, which is used to characterize point processes in statistics.^35^^,^^36^^,^^37^ The J-function is defined as $J\left(r\right)=\frac{1-G\left(r\right)}{1-F\left(r\right)}$, where the G(r) and F(r) are the nearest neighbor distribution and the spherical contact distribution, respectively. In the case of a complete random point process, the J-function is calculated as J(r)=1. Based on this value, the J-function indicates whether the points form a clustered structure or a regular structure: J(r)<1 indicates clustering and J(r)>1 indicates regularity of points. The J-function allows us to determine if the points interact repulsively or attractively. Since our topological defects can have different charges, we compute four kinds of J-functions as $J_{++}\left(r\right)=\frac{1-G_{++}\left(r\right)}{1-F_{+}\left(r\right)}$, $J_{+-}\left(r\right)=\frac{1-G_{+-}\left(r\right)}{1-F_{-}\left(r\right)}$, $J_{-+}\left(r\right)=\frac{1-G_{-+}\left(r\right)}{1-F_{+}\left(r\right)}$, and $J_{--}\left(r\right)=\frac{1-G_{--}\left(r\right)}{1-F_{-}\left(r\right)}$, where $G_{\pm \pm }\left(r\right)$ is the nearest neighbor distribution from positive (negative) defects to positive (negative) defects and $F_{\pm }\left(r\right)$ is the spherical contact distribution of positive (negative) defects. The subscripts indicate the charges of the referencing defect and the target defect. For example, $J_{+-}\left(r\right)$ represents the J-function computed by measuring the distance from a positive defect to its nearest negative defect.

Figure 4C shows the J-functions for the topological defects generated by the same process as the spin configuration in Figure 4A. Note that the graph represents the averaged J-functions, calculated from 100 independently generated spin configurations. The J-functions for identical charge defect pairs, $J_{++}\left(r\right)$ and $J_{--}\left(r\right)$, are larger than 1 for all distances, indicating that they interact repulsively. The J-function for opposite charge defect pairs, $J_{+-}\left(r\right)$ and $J_{-+}\left(r\right)$, are larger than 1 in the region of $r<\frac{\pi J}{D}$, indicating repulsive interactions at short range. Conversely, in the long-range ($r>\frac{\pi J}{D}$), the J-function is smaller than 1, indicating an attractive interaction between opposite charge defects. As a result, opposite charge pairs maintain the characteristic length, $\frac{\pi J}{D}$. Figure 4D shows the topological defects with additional circles indicating their effective range, each with a radius of $\frac{\pi J}{2D}$. The effective ranges of opposite charges tend to contact each other, while those with identical charges do not.

Figures 4E–4H shows a similar result for a spin configuration generated under different simulation settings. An external field is applied in the early steps of the simulation to help the spin configuration align along the field direction. As a result, the final spin configuration contains fewer topological defects compared to that in Figure 4A. Despite the differing number of topological defects, their behavior is similar, showing analogous interaction characteristics: identical charge defects interact repulsively, and opposite charge defects maintain the characteristic length, $\frac{\pi J}{2D}$.

### Application: Analyzing experimental data and expanding the methodology

**Figure 5:**
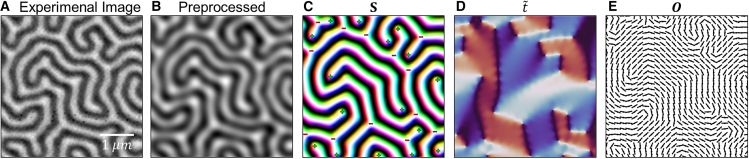
Application of our approach to experimental data (A) Experimental image of a magnetic domain. (B) Gaussian blurred version of (A). (C) Spin configuration S constructed using a greedy algorithm from the initial condition in (B). (D) The order parameter $\tilde{t}$ and (E) the orientation field O corresponding to the spin configuration in (C).

We further demonstrate an application of our method by analyzing topological defects in experimental data. Figure 5A presents a scanning transmission X-ray microscopy (STXM) image^38^ of a magnetic domain in a [Pt(3 nm)/GdFeCo(5 nm)/MgO(1 nm)]_20_ multilayer system, where the Pt underlayer introduces a strong DM interaction.^7^ This system exhibits a complex maze-like structure with numerous endpoints and three-way junctions in the out-of-plane region.

Since the experimental image directly provides only the out-of-plane component, we construct the full spin configuration from the experimental data in two steps. First, we reduce its noise using Gaussian blurring (Figure 5B). Second, we construct the full spin configuration through a greedy algorithm,^39^ i.e., aligning spin direction along the effective field predicted by our Hamiltonian model (which, in our case, is based on exchange interaction and Bloch-type DM interaction). It must be noted that this approach does not reconstruct the true in-plane spin directions, which would require additional measurements or detailed physical modeling. The actual experimental system is a ferrimagnetic film whose in-plane spin texture follows a Néel-type configuration. Despite the difference between the true spin structure and the one we constructed under the assumption of Bloch-type DM interaction, our analysis based on the magnetic structure tensor, the derived order parameter, and the defect identification remains valid. This is because the difference introduces only a constant phase factor ($\theta =\frac{2}{\pi }$) in the order parameter $\tilde{t}=\left\{\chi_{\alpha },\chi_{\beta }\;\right\}$, as described in Equation 15, which does not affect the topological classification of defects.

Figure 5C shows the reconstructed spin configuration along with the identified topological defects. The topological defects are identified using our order parameter, with positive defects located at endpoints and negative defects found at junctions, consistent with our simulation results. Figures 5D and 5E display the corresponding order parameter $\tilde{t}$ and orientation field O obtained by our method. We confirm that our method successfully identifies endpoints and three-way junctions as the defects in the maze-like experimental data. This approach has multiple applications, including the detection of stripe pattern mismatches that can be identified as topological dipole or quadrupole defects.

**Figure 6:**
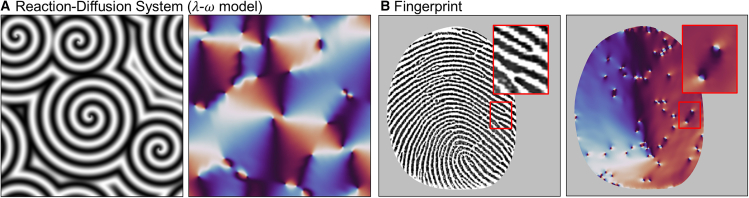
Applying methodologies beyond the magnetic domain (A and B) Our method on (A) a spiral wave pattern of the λ*-*ω model in a reaction-diffusion system and (B) a contact-based fingerprint image from *The Hong Kong Polytechnic University Contactless 2D to Contact-based 2D Fingerprint Images Database Version 1.0*. Each $\tilde{t}$ field (right figure) identifies the orientation of patterns and the locations of topological defects in the original image (left figure).

To demonstrate further applications of our approach, we examine it in two other domains: the λ*-*ω model in reaction-diffusion system^40^ and a contact-based fingerprint image. Figure 6A illustrates a typical spiral wave pattern generated by the λ*-*ω model and its corresponding $\tilde{t}$ field. We interpret the wave pattern into the out-of-plane spin components and build the full spin configuration through the same approach as in Figure 5 (use the out-of-plane magnetization as the initial condition of the greedy algorithm). Each spiral in the image is transformed into a radial structure in the $\tilde{t}$, featuring two positive defects at the center of the spiral. These defects represent the endpoints at the center of the spiral, demonstrating how our approach effectively converts complex wave structures into topological representations.

Figure 6B presents a contact-based fingerprint image^41^ and its corresponding $\tilde{t}$ field. The overall fingerprint structure is characterized by periodic ridge-valley patterns, which globally exhibit a 180° rotation. This global rotation is consistently captured in the $\tilde{t}$ field, demonstrating the effectiveness of our approach in identifying and preserving the large-scale structural features of the fingerprint. In addition to the global structure, the fingerprint contains multiple discontinuities, such as ridge endings, which correspond to localized topological defects in the $\tilde{t}$ field. The zoomed-in boxes highlight a quadrupole defect structure in the $\tilde{t}$ field, which is topologically equivalent to the spin configurations shown in Figures 3B–3(iii). We confirm that our approach successfully captures both global and local topological features, enabling a comprehensive analysis of complex patterns.

### Conclusion

We introduced the magnetic structure tensor as a tool for characterizing topological defects in two-dimensional magnetic textures. Our approach successfully identified topological monopoles, dipoles, and quadrupoles using the derived order parameter. This method provides insights into defect distribution and interactions, confirmed by both simulations and experimental data. Energetic analysis showed that the topological monopoles and dipoles exhibit delocalized energy, while quadrupoles are localized. Pairwise distance analysis using the J-function revealed charge-dependent interactions between defects. Furthermore, we applied our method to STXM images, the λ*-*ω model in reaction-diffusion systems, and fingerprint patterns. In each case, the method successfully identified the respective topological defects. These results confirm the proposed approach provides an effective framework for analyzing topological features and identifying topological defects not only in magnetic systems but also across a wide range of complex patterns.

### Limitations of the study

Our previous analysis assumes that the Bloch-type DM interaction dominates the system and relies on the spatial variations of the spin configuration. In real materials, however, magnetic domains can be uniform over large areas, leading to negligible spin gradients, (i.e., $\chi_{ij}\approx 0$), in which case our approach is not applicable. Furthermore, contrary to the assumed simple in-plane spin directions, magnetic domain walls may host additional excitations—such as domain wall skyrmions^42^^,^^43^ or Bloch points^44^ (three-dimensional singularity)—that are not captured by our current method. Therefore, to perform topological analysis in such complex systems, further experiments and a more advanced theoretical framework may be necessary.

## Resource availability

### Lead contact

Further information and any requests should be directed to and will be fulfilled by the lead contact, Prof. Changyeon Won (cywon@khu.ac.kr).

### Materials availability

This study did not generate new unique reagents.

### Data and code availability

The fingerprint image used in this study was obtained from *The Hong Kong Polytechnic University Contactless 2D to Contact-based 2D Fingerprint Images Database Version 1.0* (available with permission at “https://www4.comp.polyu.edu.hk/∼csajaykr/fingerprint.htm”). This paper does not report the original code. Any additional information required to reanalyze the data reported in this paper is available from the lead contact upon request.

## Acknowledgments

This research was supported by the 10.13039/501100003725National Research Foundation (10.13039/501100003725NRF) of Korea funded by the Korean Government (NRF-2021R1C1C2093113, NRF-2023R1A2C1006050, and RS-2024-00451261); and by the Korea Institute of Science and Technology Institutional Program (2E32951).

## Author contributions

S.M.P. devised algorithms, generated the simulation data, and experimented with the algorithms; T.J.M., G. Y., H.G.Y., H.Y.K., and C.W. contributed to the discussions of the main results; J.W.C. provided the experimental data; H.Y.K. and C.W. equally supervised the work progress. All authors contributed to the final manuscript.

## Declaration of interests

The authors declare no competing interests.

## Declaration of generative AI and AI-assisted technologies in the writing process

During the preparation of this work the authors used *ChatGPT* of OpenAI and *AI Studio* of Google, in order to develop batter readability. After using these tools, the authors reviewed and edited the content as needed and take full responsibility for the content of the publication.

## STAR★Methods

### Key resources table

**Table:** 

REAGENT or RESOURCE	SOURCE	IDENTIFIER
**Software and algorithms**		

Blender	Blender Foundation	https://www.blender.org/;RRID:SCR_008606
Reaction-diffusion system simulation	VisualPDE	https://visualpde.com/sim/?preset=lambdaOmega

**Other**		

Fingerprint Image	The Hong Kong Polytechnic University	https://www4.comp.polyu.edu.hk/∼csajaykr/fingerprint.htm

### Method details

The simulation results in Figures 2, 3, 5, and 6 were obtained using an effective field greedy algorithm. For the spin configurations in Figures 2 and 3, we prepared initial spin configurations on a 1260 × 1260 lattice with all spins pointing either upward or downward which are expected to be released into typical magnetic structures. In the greedy algorithm step, the effective field at each site is computed based on the Hamiltonian described in the manuscript, and the spin configuration is updated by aligning each spin along its local effective field direction. The greedy algorithm was repeated 100,000 iterations. For Figures 5 and 6, we used Hamiltonian parameters of J=1.0 and D=0.3. The initial spin configuration was prepared by resizing the target images (experimental image, reaction-diffusion image, or fingerprint image) by a specific ratio, so that its length scale roughly matched the characteristic length scale set by the Hamiltonian parameters. The greedy algorithm for these spin configurations was repeated 100 iterations.

The simulation results in Figure 4 are obtained through Metropolis–Hastings algorithm. Specifically, we used a 512×512 lattice and performed the Monte Carlo simulation by linearly decreasing the simulation temperature from 1 to 0 over 100,000 iterations. For the spin configuration in Figure 4A, we use the same Hamiltonian model described in the manuscript, with Hamiltonian parameters of J=1.0 and D=0.3. The spin configuration in Figure 4E used the same parameters but included an additional external field with ${\vec{H}}_{\text{ext}}=\left(\frac{0.05}{\sqrt{2}},\frac{0.05}{\sqrt{2}},0\right)$. After the Monte Carlo simulation, the spin configurations were additionally relaxed using the effective field greedy algorithm for 10,000 iterations, using the Hamiltonian model without any external field.

The spiral wave pattern shown in Figure 6A is generated through λ–ω model. The dynamics are described by the following coupled equations: $\frac{\partial u}{\partial t}=0.2\nabla^{2}u+3u-\left(u-v\right)\left(u^{2}+v^{2}\right),\frac{\partial v}{\partial t}=\nabla^{2}v+3v-\left(v-u\right)\left(u^{2}+v^{2}\right)$, where the specified parameter values have been used. The resulting scalar field u was used both to visualize the spiral wave pattern and to construct the initial spin configuration.
